# Can Trauma Condition Vulnerable Individuals to Develop Catatonic Symptoms?

**DOI:** 10.3390/brainsci10060354

**Published:** 2020-06-08

**Authors:** Jackson Hortenstine, Nagy Youssef

**Affiliations:** 1 Medical College of Georgia at Augusta University, 997 St. Sebastian Way, Augusta, GA 30912, USA; jhortenstine@augusta.edu; 2 Department of Psychiatry and Health Behavior & Office of Academic Affairs, Medical College of Georgia at Augusta University, 997 St. Sebastian Way, Augusta, GA 30912, USA

**Keywords:** conditioning, catatonia, post-traumatic stress disorder

## Abstract

Limited research has been done on the risk and predisposing factors of catatonic symptoms induced by traumatic events. There seem to be two types or constructs of conditioning that predispose an individual to catatonic symptoms in response to traumatic events: external conditioning and internal conditioning. Here, we review a study that found a significant correlation between the Bush–Francis Catatonia Scale and the Adverse Childhood Experience questionnaire; we also review studies of rats that were conditioned to expect an electric shock who developed catatonia-like immobility without the shock being applied. We also review the clinical case report of a previously traumatized individual.

Limited research has been done on the risks and predisposing factors of catatonia due to traumatic events. Here, we will discuss the emerging research on conditioning that precipitates catatonic symptoms after a traumatic event. A study using the Bush–Francis Catatonia Scale (BFCS) questionnaire showed that in 100 participants currently receiving in-patient care in a trauma program that provides group and individual counseling, the average BFCS score was 7.7 (SD = 10.3); 81 participants scored 2 or higher and 67 scored 5 or higher [1]. The same population were given the Adverse Childhood Experience (ACE) questionnaire, which showed 91 participants had a score of 4 or greater. They found a significant correlation between BFCS scores and ACE questionnaire scores, which suggests that stress or trauma during childhood could condition or predispose a person to develop catatonic symptoms [1]. This is the first study to examine the relationship between adverse childhood experiences and catatonia on a somewhat large scale. Another study in mice has shown that when placed in conflict situations, mice react differently to certain stimuli based on if they were chosen to continually win or lose in the conflicts. When the mice were pinched on the scruff of the neck, the consistent losers spent more time in a catatonia-like state while the consistent winners spent much less time in this catatonia-like state [2]. These results may add to the accumulating evidence that trauma may condition an organism to the development of catatonia-like states to varying degrees, based on prior vulnerability.

There seem to be two types or constructs of conditioning that predispose an individual to catatonic symptoms in response to traumatic events: external conditioning (e.g., external environment) and internal conditioning (e.g., flashbacks or nightmares).

As an example of external conditioning, a basic scientific study showed that rats which were conditioned to expect an electric shock when placed in a test box developed catatonia-like immobility and had electroencephalogram (EEG) changes in the cortex and hippocampus even without the shock being applied. Once put back in their home cage, their behavior and EEG normalized. The catatonic behaviors and EEG changes reappeared immediately on reintroduction to the test box. When the rats were pretreated with diazepam, nicotinamide, and chlorpromazine, then placed in the test box, they had less of a catatonic response [3]. In this case, the electric shock was the external force that seemed to prime the catatonic symptoms in the rats.

An example of internal conditioning could be flashbacks of a traumatic experience, as described in a case study which attributed the onset of catatonia in a 21-year-old woman to her four years spent as a captive of a cult, during which she experienced severe psychological, physical, and sexual abuse. According to her family, she had no mental health issues prior to her captivity. The authors reported, “Her initial symptoms were fear and startle responses on encountering loud stimuli and flashbacks in which she would lose all awareness of her current surroundings and speak about what occurred in the motel” [4]. It seems that these flashbacks were brought on by the internal thoughts that pulled her back into the motel, and she developed catatonic symptoms that were primed during her captivity. A 1 mg lorazepam challenge was attempted and was successful as an initial treatment for her catatonic state. The authors do admit that while it is likely the patient’s catatonia was caused by her experiences while in captivity, they go on to state, “Unfortunately there are no systematic studies or estimates of the number of cases in which these factors play a role” [4]. Dunn states, “It is essential that patients who present with symptoms of catatonia be screened for trauma” [4].

More research on the correlation between trauma and catatonic symptoms needs to be done in order to investigate and replicate the role of conditioning and priming in inducing catatonic symptoms in traumatized patients as a first step to further understand these relationship and develop better therapeutics.
